# Pathological depositions in human disease: converging mechanisms in atherosclerosis, Alzheimer's disease, and related disorders

**DOI:** 10.1186/s43556-026-00552-y

**Published:** 2026-08-20

**Authors:** Louis Ragolia

**Affiliations:** Department of Foundations of Medicine, NYU Grossman Long Island School of Medicine, 101 Mineola Blvd, Ste. 4-004, Mineola, NYU 11501 USA

**Keywords:** Pathological deposition, Atherosclerosis, Alzheimer’s disease, NLRP3 inflammasome, Apolipoprotein A-I, Lysosomal storage disorders

## Abstract

Pathological deposition of endogenous material within tissues represents a central, unifying mechanism across a broad spectrum of prevalent human diseases. Atherosclerosis and Alzheimer's disease (AD) exemplify this paradigm: atherosclerosis is driven by subendothelial accumulation of apolipoprotein B–containing lipoproteins, cholesterol crystals, and hydroxyapatite, while AD is characterized by cerebral deposition of misfolded amyloid-β (Aβ) and/or hyperphosphorylated tau. Emerging evidence implicates substantial lipid dyshomeostasis in AD pathogenesis, including lipid-droplet-accumulating microglia and widespread lipidomic disruption, blurring the categorical boundary between "lipid" and "protein" deposition diseases. Apolipoprotein A-I (apoA-I) dysfunction bridges both domains: post-translational modifications and point mutations impair reverse cholesterol transport in atherosclerosis and simultaneously promote apoA-I amyloid fibril formation in systemic amyloidosis. Across atherosclerosis, AD, systemic amyloidoses, immune complex–mediated nephropathies, crystal arthropathies, and lysosomal storage disorders, shared pathophysiologic processes emerge: production–clearance imbalance, conformational transitions favoring aggregation or crystallization, microenvironmental modulation by extracellular matrix components, and chronic sterile inflammatory responses driven by NLRP3 inflammasome activation. In this review, we synthesize deposition biology using atherosclerosis and AD as primary paradigms, integrate mechanistic insights from related disorders, highlight convergent molecular pathways including NLRP3, proteostasis, and glymphatic clearance, and discuss diagnostic and therapeutic strategies. A conceptual framework unifying these conditions as variations on a common deposition theme is proposed to guide broadly applicable precision therapies.

## Introduction

Deposition of abnormal material within tissues is a defining feature of many prevalent and debilitating human diseases [1–4]. Although these conditions are traditionally approached within organ-specific subspecialties such as cardiology, neurology, nephrology, and rheumatology, they share several core pathophysiologic principles: dysregulated production or aberrant generation of specific molecules or particles, impaired clearance, structural transitions that favor aggregation or crystallization, and microenvironmental conditions that facilitate accumulation and persistence [5–8]. These processes consistently elicit chronic inflammation, tissue remodeling, and progressive organ dysfunction, ultimately converging on clinical endpoints that impose an enormous societal burden [9].

Atherosclerosis and Alzheimer’s Disease (AD) have been selected as the focal paradigms of this review for compelling scientific and clinical reasons. First, they represent the leading causes of cardiovascular mortality and dementia worldwide, together accounting for an outsized proportion of age-related morbidity and mortality [10, 11]. Second, both conditions involve molecularly well-defined deposits that enable detailed mechanistic interrogation: subendothelial apolipoprotein B (apoB)–containing lipoproteins and cholesterol crystals in atherosclerosis [12–14], and extracellular Aβ plaques together with intraneuronal and/or extracellular tau neurofibrillary tangles in AD [15–17]. Third, and most importantly, a substantial and growing body of epidemiological and mechanistic evidence reveals bidirectional links between vascular and neurodegenerative pathology that go beyond coincidental association, suggesting that these two diseases share specific molecular nodes, including APOE, NLRP3, apoA-I, efferocytosis, and glymphatic clearance pathways, amenable to co-targeting [18–24]. This unique convergence justifies their paired treatment as paradigms and provides added value not available from single-disease reviews. Other deposition diseases, including systemic amyloidoses, immune complex–mediated glomerulonephritides, crystal-induced arthropathies, and lysosomal storage disorders, further extend and validate the generalizability of this framework [25–43].

Rather than treating these disorders as isolated entities, this review is organized around a unifying conceptual framework: pathological deposition emerges when production of a potentially harmful material exceeds the capacity of cellular, vascular, or lysosomal systems to clear, sequester, or remodel it. In this framework, atherosclerosis and AD serve as complementary paradigms. One is centered on lipid retention and crystallization and the other on protein misfolding and aggregation, and both are shaped by shared mechanisms including defective clearance, extracellular matrix stabilization, innate immune activation, and chronic remodeling. The sections that follow therefore move from this convergence framework to the common molecular logic of deposition, then to the two primary paradigms, and finally to related disorders, shared pathways, diagnostics, therapeutics, and translational implications. This structure is intended not as a catalogue of diseases, but as a synthesis of how diverse deposition disorders can be understood as variations on a common biological theme.

## Convergence of vascular and neurodegenerative deposition: an organizing framework

The convergence of vascular and neurodegenerative pathology represents the conceptual foundation upon which this review is built. Introducing this framework at the outset, rather than as a concluding observation, provides readers with a roadmap for understanding why mechanistic parallels between atherosclerosis and AD reflect shared biology with direct therapeutic implications.

Epidemiological studies demonstrate robust associations between vascular risk factors such as hypertension, dyslipidemia, type 2 diabetes, obesity, and smoking, and the risk of cognitive decline, AD, and dementia [44–46]. The 2020 Lancet Commission on dementia prevention identified 12 modifiable risk factors, many of them cardiovascular, that together account for approximately 40% of dementia cases worldwide [20]. Cerebral small vessel disease, atherosclerosis of the circle of Willis, and cerebral amyloid angiopathy (CAA) frequently coexist with AD neuropathology, contributing to mixed dementias and accelerating cognitive decline [19, 21, 22]. These observations reflect shared pathways including oxidative stress, endothelial dysfunction, impaired clearance of neurotoxic proteins via vascular and glymphatic routes, and chronic low-grade inflammation [47–51].

The vascular hypothesis of AD proposes that cerebrovascular dysfunction and impaired perivascular drainage promote Aβ accumulation in brain parenchyma and vessel walls [51–55]. Conversely, Aβ and tau deposits disrupt the neurovascular unit and blood–brain barrier (BBB) integrity, further impairing vascular function [56]. This bidirectional interplay, the "vascular-amyloid continuum", suggests that optimizing vascular health may yield neuroprotective benefits and that insights from AD biology may inform vascular interventions. Blood–brain barrier breakdown has been identified as an early, pericyte-mediated biomarker of cognitive dysfunction that precedes overt Aβ and tau pathology [21]. Reduced cerebral blood flow, pericyte loss, and impaired perivascular clearance are additional vascular contributors that operate synergistically with amyloid and tau pathology [57]. Emerging evidence identifies novel therapeutic targets within the neurovascular unit that may slow disease progression by simultaneously addressing vascular and protein deposition mechanisms [58].

**Fig. 1:**
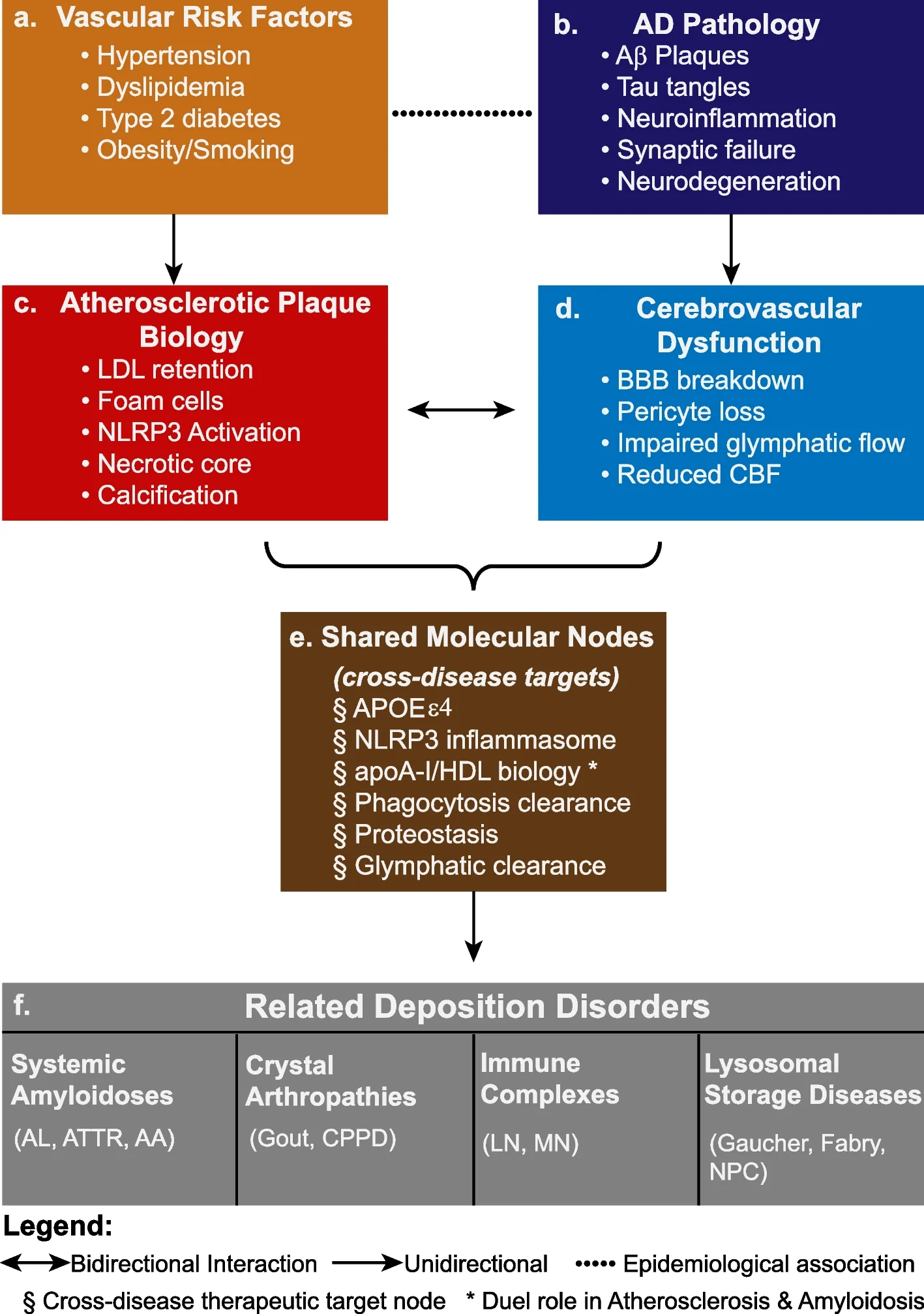
The vascular–amyloid continuum and shared deposition mechanisms across atherosclerosis, Alzheimer’s disease, and related disorders. Schematic overview of the vascular–amyloid continuum illustrating the relationships between vascular risk factors, atherosclerotic plaque biology, cerebrovascular dysfunction, and Alzheimer’s disease (AD) pathology. Vascular risk factors, including hypertension, dyslipidemia, type 2 diabetes, and obesity/smoking, promote atherosclerotic plaque development and are epidemiologically associated with AD. Atherosclerotic plaque biology and cerebrovascular dysfunction interact bidirectionally through shared mechanisms including endothelial injury, inflammatory activation, impaired perfusion, and defective solute clearance. These processes converge on a set of shared molecular nodes- APOE ε4, NLRP3 inflammasome signaling, apoA-I/HDL biology, efferocytosis/phagocytosis, proteostasis networks, and glymphatic clearance, that represent potential cross-disease therapeutic targets. The related deposition disorders shown below the shared-node box emphasize the broader applicability of this framework to systemic amyloidoses, crystal arthropathies, immune complex diseases, and lysosomal storage disorders. The asterisk indicates the dual role of apoA-I/HDL biology in both atherosclerosis and amyloidosis

At the molecular level, apolipoprotein E (APOE), particularly the ε4 allele, is the strongest genetic risk factor for sporadic AD and influences lipoprotein metabolism and atherosclerosis risk, providing a shared molecular bridge [59, 60]. A further dimension of convergence involves apolipoprotein A-I (apoA-I), whose post-translational modifications and point mutations simultaneously impair reverse cholesterol transport, promoting atherosclerosis, and induce misfolding and deposition as amyloid fibrils in multiple organs [61–64]. ApoA-I amyloidosis thus sits at the intersection of lipid metabolism and protein aggregation diseases, further reinforcing the conceptual unity of the deposition paradigm. These relationships are summarized in Fig. 1.

## Classes and principles of pathological deposition

Once the vascular–amyloid continuum is established as the organizing framework, the next step is to ask what all deposition diseases are built from and what rules govern their accumulation. Although the deposited materials differ in composition, ranging from retained lipoproteins and misfolded proteins to immune complexes, crystals, and incompletely degraded lysosomal substrates, they can be understood through a common set of biological principles that determine whether a molecule remains soluble, is cleared efficiently, or becomes retained and pathogenic. The categories described below are therefore not meant simply to classify diseases by composition, but to clarify how distinct materials enter a shared pathogenic logic of persistence, inflammatory activation, and tissue injury.

### Classes of deposited material

Pathological deposits can be categorized by composition and localization. The categories below are not mutually exclusive; many advanced deposition diseases involve multiple deposit types simultaneously, and the boundaries between "lipid" and "protein" deposition diseases are increasingly recognized as artificial, as elaborated in Sects. " Atherosclerosis: a lipid deposition paradigm" and " Alzheimer's disease: a protein deposition disorder with a lipid dimension".

#### Lipid-rich deposits

Subendothelial retention and modification of low-density lipoprotein (LDL) and triglyceride-rich remnant lipoproteins in atherosclerotic plaques [65, 66], including cholesterol crystals in advanced lesions [12, 14]. Importantly, lipid dysregulation also operates in AD: lipid-droplet-accumulating microglia (LDAM) represents a dysfunctional, pro-inflammatory state in the aging brain [67–70], and comprehensive lipidomic studies document widespread disruption of sphingolipids, phospholipids, and cholesterol esters in AD brain tissue with broad pathological implications [71].

#### Protein aggregates (amyloid and non-amyloid)

Protein aggregates such as Aβ plaques and tau tangles in AD [72–74]; transthyretin (TTR), immunoglobulin light chains, and serum amyloid A in systemic amyloidoses [75–78]; α-synuclein aggregates in synucleinopathies [79–81]; and apoA-I fibrils in hereditary apoA-I amyloidosis [82, 83].

#### Immune complexes and complement components

Glomerular and vascular immune complex deposition in lupus nephritis, membranous nephropathy, and immune complex vasculitis [84–86].

#### Mineral and crystalline deposits

Hydroxyapatite in vascular and valvular calcification [87]; monosodium urate crystals in gout [88]; calcium pyrophosphate dihydrate (CPPD) in pseudogout [89]; calcium oxalate and other crystals in nephrolithiasis [90].

#### Metabolic storage products

Glycosphingolipids, glycosaminoglycans, and other substrates are observed in lysosomal storage diseases [91–95] and glycogen in glycogen storage disorders [96, 97].

Despite compositional heterogeneity, these deposits share common pathophysiologic themes outlined in Sect. " Shared pathophysiologic principles". The convergent features are far more instructive than the differences and understanding them is essential for identifying shared therapeutic targets.

### Shared pathophysiologic principles

Across deposition disorders, five recurring processes are observed (summarized in Table 1):

**Table 1:** Comparative features of major deposition disorders across composition, localization, pathogenic mechanisms, clearance, inflammasome involvement, and representative therapeutic strategies

Disease	Deposited material	Primary localization	Core pathogenic mechanism	Main clearance/homeostatic pathway(s)	NLRP3 involvement	Representative therapies	Key references
Atherosclerosis	ApoB-containing lipoproteins; cholesterol crystals; hydroxyapatite	Arterial intima; fibrous cap; necrotic core	Lipoprotein retention, modification, foam-cell formation, defective efferocytosis, crystal-driven inflammation	Macrophage efferocytosis; reverse cholesterol transport via apoA-I/HDL/ABCA1/ABCG1	Direct/validated	Statins; ezetimibe; PCSK9 inhibitors; inclisiran; canakinumab; colchicine; apoA-I-based approaches	[5, 6, 12, 98–100]
Alzheimer’s disease	Aβ; hyperphosphorylated tau; lipid droplets in LDAM	Brain parenchyma; cerebral vessel walls (CAA)	Amyloidogenic APP processing, impaired proteolytic/vascular/glymphatic clearance, tau propagation, glial inflammatory amplification	Microglial phagocytosis; glymphatic/perivascular drainage; LRP1-mediated BBB efflux; neprilysin/IDE degradation	Direct/preclinical	Lecanemab; donanemab; anti-tau approaches; investigational NLRP3 inhibitors	[10, 11, 47, 51, 67, 71, 101–104]
AL amyloidosis	Monoclonal immunoglobulin light chains	Heart; kidneys; liver; peripheral/autonomic nerves	Plasma-cell overproduction of amyloidogenic light chains with extracellular fibril deposition	Phagocytic/proteostatic clearance	Limited/indirect	Daratumumab-based regimens; proteasome inhibitors; alkylator-based therapy	[28, 36, 78, 105]
ATTR amyloidosis	Transthyretin (wild-type or mutant)	Heart; peripheral/autonomic nerves; carpal tunnel	Tetramer dissociation, monomer misfolding, amyloid fibrillogenesis	Phagocytic/proteostatic clearance	Limited/indirect	Tafamidis; acoramidis; patisiran; vutrisiran; eplontersen	[30, 35, 106–108]
AA amyloidosis	Serum amyloid A	Kidneys; liver; spleen; adrenals	Chronic inflammatory overproduction of SAA followed by fibril deposition	Phagocytic/proteolytic clearance	Indirect	Control of underlying inflammatory disease; IL-1, IL-6, or TNF pathway inhibition	[38, 76, 109]
ApoA-I amyloidosis	ApoA-I fibrils	Liver; kidneys; peripheral nerves; skin	APOA1 mutation- or modification-driven structural destabilization and fibril formation	Phagocytic clearance	Under investigation	ApoA-I mimetics; recombinant HDL-related strategies; investigational chaperone approaches	[61, 63, 64, 82, 83, 110]
Gout	Monosodium urate crystals	Joints; periarticular tissues; tophi	Hyperuricemia with crystal precipitation and IL-1β-driven sterile inflammation	Phagocytosis; renal urate excretion	Direct/validated	Colchicine; urate-lowering therapy; IL-1 inhibitors	[26, 34, 88, 111]
CPPD disease	Calcium pyrophosphate dihydrate crystals	Articular cartilage; synovium	CPPD crystal deposition with crystal-triggered innate immune activation	Phagocytosis	Direct/analogous	Colchicine; NSAIDs; IL-1 inhibition off-label	[41, 89]
Lupus nephritis	Immune complexes and complement	Glomeruli; tubular basement membrane	Autoimmune immune-complex deposition with complement-mediated inflammatory injury	Mesangial/phagocytic and complement-dependent clearance	Indirect	Hydroxychloroquine; mycophenolate; belimumab; voclosporin	[25, 42, 86, 112]
Membranous nephropathy	Subepithelial immune complexes, often anti-PLA2R-associated	Glomerular basement membrane	In situ immune-complex formation with complement-mediated podocyte injury	Local immune-complex handling and complement regulation	Indirect	Rituximab; cyclophosphamide; calcineurin inhibitors; obinutuzumab	[40, 85, 113]
Immune complex vasculitis	IgG/IgA/cryoglobulin-containing immune complexes	Small- to medium-vessel walls	Immune-complex deposition with leukocytoclastic vascular inflammation	Phagocytic and complement-mediated clearance	Indirect	Corticosteroids; rituximab in selected forms; treatment of underlying cause	[33, 84, 114]
Gaucher disease	Glucosylceramide (glucocerebroside)	Macrophages in spleen, liver, bone marrow; CNS in neuronopathic forms	Lysosomal enzyme deficiency with intracellular lipid storage and secondary inflammatory activation	Lysosomal catabolism (deficient); autophagy	Secondary	Enzyme replacement therapy; substrate reduction therapy	[31, 39, 115, 116]
Fabry disease	Globotriaosylceramide (Gb3)	Vascular endothelium; podocytes; cardiomyocytes; peripheral neurons	α-Galactosidase A deficiency with lysosomal glycosphingolipid accumulation	Lysosomal catabolism (deficient)	Secondary/indirect	Agalsidase alfa/beta; migalastat	[29, 39]
Niemann–Pick disease type C	Cholesterol; sphingolipids	Multiple tissues; CNS prominent	Defective lysosomal cholesterol/sphingolipid trafficking with neurodegenerative stress	Lysosomal trafficking/catabolism (deficient); autophagy	Secondary	Miglustat; investigational chaperone/stress-response approaches	[39, 91, 117]
Mucopolysaccharidoses	Glycosaminoglycans	Multisystem; skeleton, heart, CNS	Lysosomal enzyme deficiency with progressive intracellular substrate storage	Lysosomal catabolism (deficient)	Secondary	Enzyme replacement therapy; hematopoietic stem cell transplantation in selected subtypes	[37, 39]
Vascular calcification	Hydroxyapatite; calcium phosphate	Arterial media and intima; cardiac valves	Osteogenic transdifferentiation, matrix vesicle release, mineral nucleation, inflammatory remodeling	Matrix remodeling; limited phagocytic removal	Indirect/secondary	Phosphate-lowering strategies; vitamin K pathway-related approaches; investigational calcification inhibitors	[27, 32, 87, 90, 118, 119]

*Abbreviations*: *Aβ* amyloid-β, *AL* immunoglobulin light chain, *APOE* apolipoprotein E, *APP* amyloid precursor protein, *apoA-I* apolipoprotein A-I, *AQP4* aquaporin-4, *ATTR* transthyretin amyloidosis, *BACE1* β-site APP cleaving enzyme 1, *BBB* blood–brain barrier, *CAA* cerebral amyloid angiopathy, *CPPD* calcium pyrophosphate dehydrate, *DAM* disease-associated microglia, *ERT* enzyme replacement therapy, *GBA1* glucocerebrosidase gene, *HDL* high-density lipoprotein, *IDE* insulin-degrading enzyme, *IL* interleukin, *LDAM* lipid-droplet-accumulating microglia, *LDL* low-density lipoprotein, *LRP1* low-density lipoprotein receptor-related protein 1, *MSU* monosodium urate, *NFT* neurofibrillary tangle, *NLRP3* NOD-like receptor protein 3, *NPC* Niemann-Pick disease type C, *PLA2R* phospholipase A2 receptor, *PCSK9* proprotein convertase subtilisin/kexin type 9, *RCT* reverse cholesterol transport, *SAA* serum amyloid A, *SRT* substrate reduction therapy, *TREM2* triggering receptor expressed on myeloid cells 2, *TTR* transthyretin, *UPS* ubiquitin–proteasome system, *VSMC* vascular smooth muscle cell

NLRP3 evidence grading: "Yes" = direct mechanistic evidence with in vivo validation; "Indirect" = downstream inflammatory pathway engagement without direct NLRP3 complex assembly demonstrated; "Secondary" = lysosomal permeabilization-dependent; "Limited evidence" = insufficient or inconsistent evidence in current literature; "Under investigation" = active preclinical or translational research ongoing

Overproduction or aberrant generation: Hyperlipidemia and increased apoB lipoproteins drive atherosclerosis. Abnormal amyloid precursor protein (APP) is a type-I transmembrane glycoprotein expressed on neuronal and non-neuronal cell surfaces, whose physiological functions include synaptic plasticity modulation and iron export [120]. APP processing generates aggregation-prone Aβ42 in AD through sequential cleavage by β-secretase (BACE1) [121] and the presenilin-containing γ-secretase complex [122]. Presenilins (PSEN1 and PSEN2) are the catalytic subunits of γ-secretase; mutations in these proteins cause familial AD by shifting the Aβ42/Aβ40 ratio toward longer, more aggregation-prone species [122, 123], while clonal plasma cells overproduce amyloidogenic light chains in AL amyloidosis [105].

#### Impaired clearance and homeostasis

Defective efferocytosis of apoptotic foam cells in advanced plaques promotes necrotic core expansion [98]. Reduced Aβ clearance via proteolytic degradation by neprilysin and insulin-degrading enzyme, BBB transport mediated by LRP1, perivascular/glymphatic drainage, and microglial phagocytosis contributes to cerebral Aβ accumulation [124–128]. Sleep-dependent glymphatic clearance is particularly important, as the glymphatic system drives metabolite clearance during sleep and sleep disruption accelerates Aβ accumulation [129, 130], while lysosomal dysfunction and defective autophagy underlie intracellular storage in lysosomal diseases [131–135].

#### Conformational change and aggregation or crystallization

Protein misfolding into cross-β amyloid fibrils (Aβ, tau, TTR, light chains, apoA-I) [15, 35, 36, 38, 61, 63, 75]. Oxidation and aggregation of LDL within the arterial intima [136, 137]. Crystallization of cholesterol and urate from supersaturated microenvironments [12, 14, 26, 34].

#### Microenvironmental modulation

Local pH, oxidative state, ionic composition, and shear stress shape deposition propensity and stability [9, 12, 14, 27]. Extracellular matrix (ECM) components such as arterial proteoglycans binding apoB; heparan sulfate proteoglycans enriched in amyloid deposits, all promote binding and nucleation [138–142]. Tissue-specific ECM composition contributes importantly to the organ tropism of different deposits [138, 143].

#### Inflammatory response and tissue remodeling

Deposits act as danger-associated molecular patterns (DAMPs), engaging pattern recognition receptors (PRRs) and NLRP3 inflammasomes, leading to chronic cytokine and chemokine production, matrix metalloproteinase activation, fibrosis, and structural distortion [6, 9, 50, 99, 144, 145]. The NLRP3 inflammasome is activated by cholesterol crystals in atherosclerosis [12, 50], by urate crystals in gout [34, 50, 111], and by Aβ in AD [101, 146], establishing it as a pan-deposition-disease inflammatory hub, as detailed in Sect. " Innate immunity, pattern recognition, and NLRP3 inflammasome activation".

These principles underscore that deposition diseases are not simply diseases of accumulation but of failed homeostasis; failures in the balanced systems of production, clearance, structural integrity, microenvironmental control, and inflammatory resolution.

**Fig. 2:**
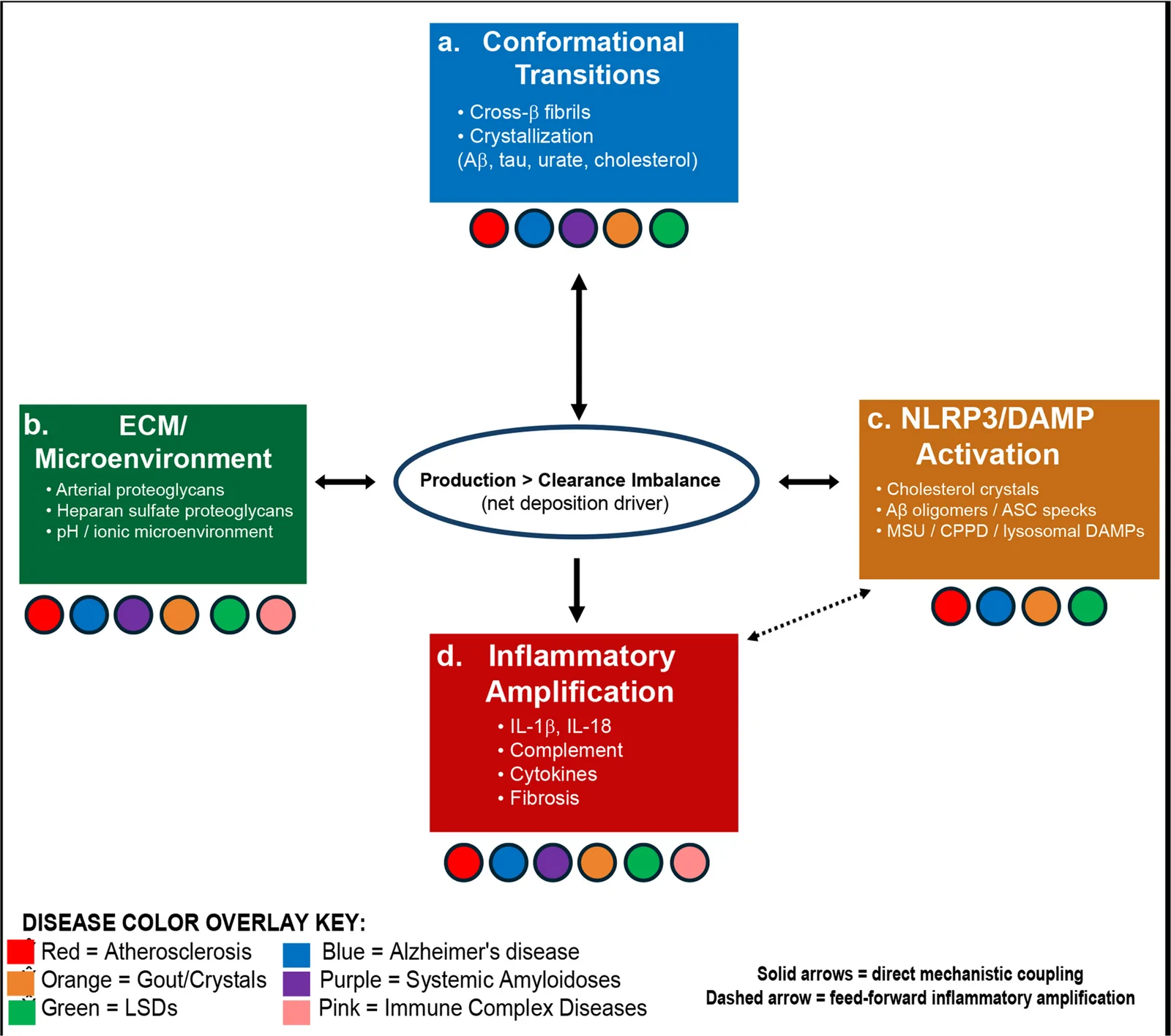
Shared pathophysiologic principles of deposition diseases. Conceptual hub-and-spoke model summarizing the shared mechanistic principles that underlie diverse deposition diseases. The central hub represents the production–clearance imbalance that drives net deposition. Four surrounding mechanistic nodes illustrate universal pathogenic processes: **a** conformational transitions favoring aggregation or crystallization, including cross-β fibril formation and crystal nucleation; **b** extracellular matrix (ECM) and microenvironmental modulation, including arterial proteoglycans, heparan sulfate proteoglycans, and local pH/ionic influences; **c** DAMP-mediated NLRP3 inflammasome and pattern-recognition receptor activation, triggered by cholesterol crystals, Aβ oligomers/ASC specks, and crystal- or lysosome-derived danger signals; and **d** downstream inflammatory amplification, including IL-1β, IL-18, complement activation, cytokine production, fibrosis, and remodeling. Solid arrows denote direct mechanistic coupling between the central deposition imbalance and the surrounding processes, whereas the dashed arrow denotes a feed-forward inflammatory amplification loop between innate immune activation and downstream inflammatory remodeling. Colored dot overlays identify disease categories engaging each mechanistic node: atherosclerosis, Alzheimer’s disease, systemic amyloidoses, crystal diseases, lysosomal storage disorders, and immune complex diseases

Viewed together, these shared principles explain why diseases that appear compositionally distinct can nevertheless follow remarkably similar pathogenic trajectories. Whether the initiating material is a retained lipoprotein, a misfolded peptide, a crystal, or an incompletely degraded lysosomal substrate, pathology emerges when production, structural transformation, and impaired clearance reinforce one another within a permissive tissue microenvironment. This framework provides the mechanistic bridge from general deposition biology to the specific vascular and neurodegenerative paradigms considered in the next two sections. The shared mechanistic framework is illustrated in Fig. 2.

## Atherosclerosis: a lipid deposition paradigm

Atherosclerosis can be viewed as the vascular archetype of pathological deposition: a disease in which a normally transported physiological cargo becomes pathogenic when retention outpaces clearance, structural transformation amplifies persistence, and inflammatory remodeling converts accumulation into tissue injury. Framed in this way, atherosclerosis is not only a disease of excess lipid, but also a model of how production–clearance imbalance, extracellular matrix interactions, defective phagocytic resolution, and sterile inflammation combine to create a clinically consequential deposit. The following subsections trace that sequence from subendothelial retention to plaque rupture while maintaining focus on the broader deposition logic shared with Alzheimer’s disease and related disorders.

### Overview and initiating events

Atherosclerosis is a chronic inflammatory disease of medium and large arteries initiated by subendothelial retention of apoB-containing lipoproteins in susceptible arterial segments [142, 147]. Endothelial dysfunction, driven by hyperlipidemia, hypertension, tobacco exposure, diabetes, and disturbed flow at branch points, increases vascular permeability and upregulates adhesion molecules including VCAM-1, ICAM-1, and selectins [5, 6]. LDL and remnant particles infiltrate the intima and bind arterial proteoglycans via electrostatic interactions with basic residues in apoB-100, prolonging residence time and creating a pro-atherogenic niche [138, 142, 147]. The response-to-retention hypothesis posits that this subendothelial lipoprotein trapping is the essential initiating event in atherogenesis [142, 147]. Optimal clinical management of atherosclerotic cardiovascular disease risk requires attention to the full spectrum of atherogenic lipoproteins including LDL, remnant lipoproteins, and Lp(a), as reflected in current guideline frameworks [100].

### Lipoprotein modification and foam cell formation

Retained lipoproteins undergo oxidative modification by reactive oxygen species (ROS) and enzymatic modification by phospholipases and sphingomyelinases, generating pro-inflammatory species including oxidized LDL (oxLDL) [136, 137]. Monocytes recruited by endothelial adhesion molecules migrate into the intima and differentiate into macrophages [5, 6, 9, 148]. Macrophages express scavenger receptors (SR-A, CD36, and LOX-1) that mediate uptake of modified LDL independently of the feedback regulation imposed by intracellular cholesterol on the LDL receptor pathway [136, 137, 148]. Cholesterol esters accumulate in lipid droplets, transforming macrophages into foam cells and forming fatty streaks, the earliest visible lesions [5, 6, 9, 113]. Under physiological conditions, cholesterol efflux via ATP-binding cassette transporters ABCA1 and ABCG1, and HDL-mediated reverse cholesterol transport, and counterbalances lipid uptake [149, 150]. This efflux pathway is critically dependent on apoA-I and functional HDL particles, as elaborated in Sect. " The role of ApoA-I, HDL, and reverse cholesterol transport". Persistent lipoprotein overload and the inflammatory milieu overwhelm these homeostatic mechanisms, driving plaque progression.

### The role of ApoA-I, HDL, and reverse cholesterol transport

ApoA-I, the principal protein component of HDL, is central to reverse cholesterol transport (RCT), the process by which cholesterol is mobilized from peripheral tissues, including arterial macrophages, and delivered to the liver for excretion [62, 64, 149]. ApoA-I mediates cholesterol efflux primarily through interaction with ABCA1, ABCG1, and SR-BI receptors on foam cells [64, 149, 151]. The molecular mechanisms governing this interaction involve apoA-I binding to ABCA1 at lipid microdomains on the macrophage surface, initiating lipid transfer and nascent HDL particle formation [151]. Disruption of these microdomains, whether by oxidative modification of apoA-I or by altered membrane lipid composition, impairs efflux efficiency [151]. Dysfunction at any step in the RCT pathway promotes intimal cholesterol accumulation and atherosclerosis [62, 64, 149].

Post-translational modifications of apoA-I, including oxidation of methionine and tryptophan residues by myeloperoxidase-derived oxidants, glycation, and carbamylation, impair its ability to promote cholesterol efflux and are enriched in atherosclerotic lesions [62, 64, 151, 152]. Myeloperoxidase-catalyzed oxidation of apoA-I at specific methionine (Met-148) and tryptophan (Trp-72) residues is demonstrably enriched in human atherosclerotic lesions and directly impairs ABCA1-mediated cholesterol efflux, establishing oxidative apoA-I modification as a key mechanism of HDL dysfunction in cardiovascular disease [152]. Point mutations in the APOA1 gene (e.g., apoA-I Milano, apoA-I Paris) can paradoxically enhance RCT and confer atheroprotection due to disulfide-linked dimer formation, whereas other mutations impair RCT and promote atherogenesis [153]. Critically, certain APOA1 mutations and post-translational modifications also destabilize the native helical structure of apoA-I, promoting its misfolding and deposition as amyloid fibrils in multiple organs [61–64, 110, 154]. ApoA-I thus exemplifies the convergence between lipid metabolism dysfunction and protein aggregation diseases, and its biology directly bridges the atherosclerosis and amyloidosis sections of this review.

### ApoA-I amyloidosis: at the intersection of vascular and protein deposition disease

Hereditary apoA-I amyloidosis is caused by mutations in the N-terminal or C-terminal regions of the APOA1 gene, which destabilize the apoA-I structure and promote fibrillogenesis [61, 63]. The native structure of apoA-I consists of a series of amphipathic α-helices organized in a helical bundle conformation; mutations in this scaffold reduce thermodynamic stability, lower the energy barrier for unfolding, and favor transition to aggregation-prone partially unfolded intermediates that nucleate fibril assembly [61, 63]. Affected individuals develop systemic amyloid deposits in the liver, kidneys, peripheral nerves, and skin. Notably, wild-type apoA-I can also be found in atherosclerotic plaques in a modified, aggregated form [62, 64, 155].

**Fig. 3:**
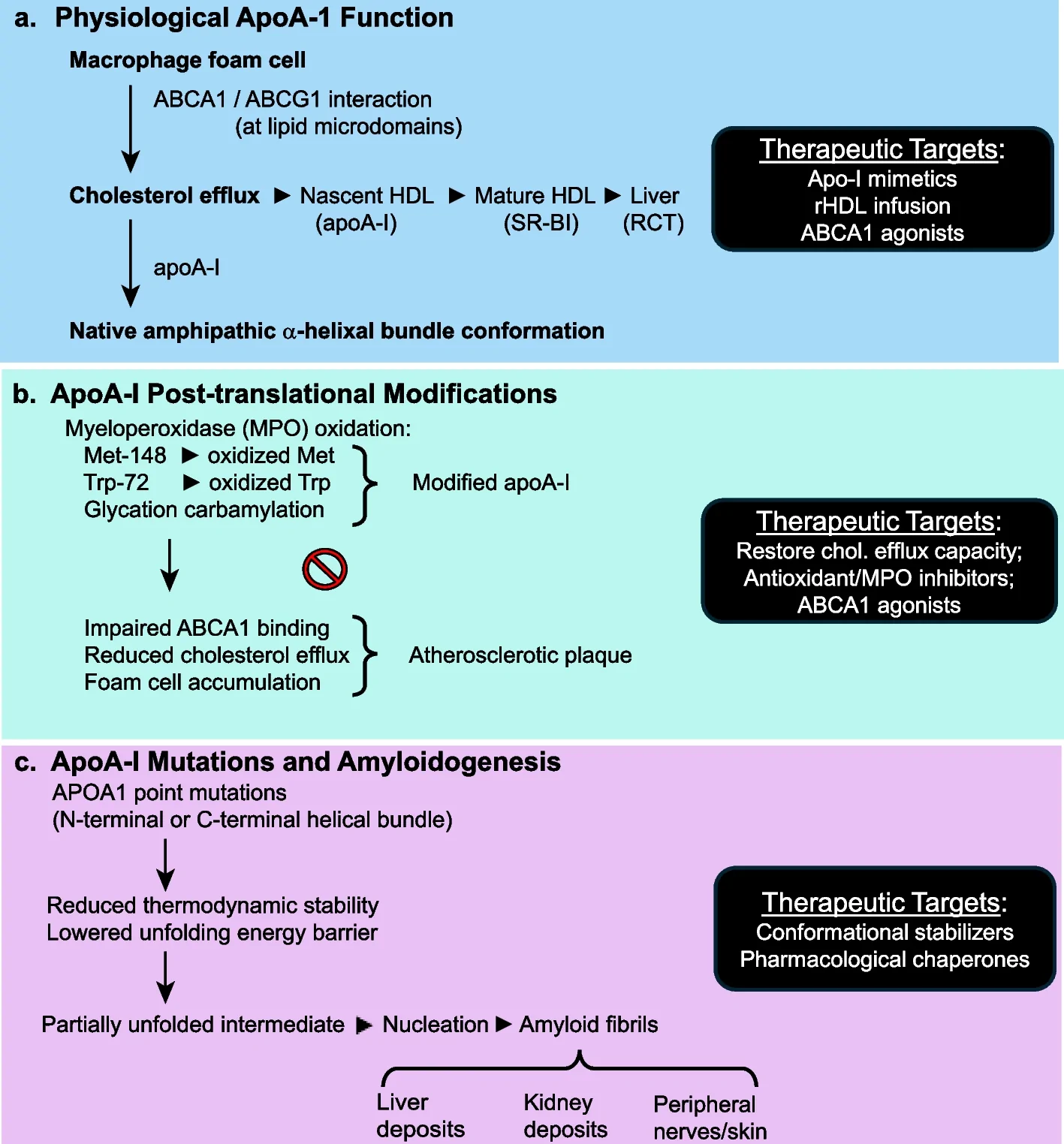
ApoA-I at the molecular intersection of atherosclerosis and amyloidosis. Three-panel schematic illustrates the dual role of apolipoprotein A-I (apoA-I) in vascular lipid homeostasis and protein aggregation disease. **a** Under physiological conditions, apoA-I promotes ABCA1/ABCG1-mediated cholesterol efflux from macrophage foam cells, supports nascent and mature HDL formation, and facilitates reverse cholesterol transport to the liver. **b** In atherosclerosis, post-translational modifications of apoA-I, including myeloperoxidase-mediated oxidation at Met-148 and Trp-72, glycation, and carbamylation, impair ABCA1-dependent cholesterol efflux, promote foam cell accumulation, and contribute to plaque progression. The inhibitory symbol highlights disruption of the apoA-I:ABCA1 interaction as a key mechanistic failure point. **c** In hereditary apoA-I amyloidosis, APOA1 point mutations destabilize the native amphipathic α-helical bundle, lower thermodynamic stability, promote partially unfolded intermediates, and drive amyloid fibril formation with deposition in the liver, kidneys, and peripheral nerves/skin. Therapeutic strategies targeting each arm of this pathway, including apoA-I mimetics, recombinant HDL, ABCA1 agonists, antioxidant/MPO-directed approaches, and conformation-stabilizing or chaperone-based strategies, are indicated

This dual role of apoA-I, as a mediator of atheroprotective RCT on one hand, and as a potential amyloidogenic protein on the other, illustrates how a single molecular dysfunction can participate in both vascular lipid deposition and systemic protein aggregation [61, 63]. Therapeutic strategies aimed at restoring apoA-I function (e.g., apoA-I mimetic peptides, recombinant HDL infusion, ABCA1 agonists) are therefore of interest in both contexts [62, 64, 156–158]. Preclinical data additionally suggest that apoA-I can solubilize and facilitate Aβ clearance, raising the possibility of neuroprotective effects in addition to cardiovascular benefits [62, 64, 159]. The dual role of apoA-I is depicted in Fig. 3.

### Defective efferocytosis and necrotic core formation

As plaques progress, macrophages and vascular smooth muscle cells (VSMCs) undergo apoptosis. Efficient efferocytosis defined as the phagocytic clearance of apoptotic cells by neighboring macrophages, is critical for inflammation resolution and plaque stability [98]. In advanced plaques, efferocytosis is impaired due to downregulation or proteolytic cleavage of efferocytosis receptors including MerTK, accumulation of anti-phagocytic "don't-eat-me" signals (CD47, CD31), and the pro-inflammatory cytokine environment [98, 160]. Uncleared apoptotic cells undergo secondary necrosis, releasing lipids and cellular contents that expand the lipid-rich necrotic core, a key determinant of plaque vulnerability [98, 160]. As discussed in Sect. " Enhancing clearance: cross-disease synergies", strategies to enhance efferocytosis in atherosclerosis are mechanistically analogous to strategies to enhance microglial phagocytosis in AD, representing a compelling shared therapeutic opportunity.

### Cholesterol crystals, NLRP3 inflammasome activation, and calcification

Cholesterol in advanced plaques can crystallize, particularly in conditions of membrane cholesterol supersaturation. Cholesterol crystals physically disrupt plaque architecture and act as potent DAMPs: they are phagocytosed by macrophages, cause lysosomal destabilization and potassium efflux, and activate the NLRP3 inflammasome, triggering caspase-1 activation and release of the pro-inflammatory cytokines IL-1β and IL-18 [12, 50, 111]. This mechanism directly links lipid deposition to sterile inflammation and plaque progression, and parallels NLRP3 activation by urate crystals in gout (Sect. " Crystal deposition diseases") and by Aβ in AD (Sect. " Glial responses, neuroinflammation, and the NLRP3 inflammasome"). The causal importance of this inflammatory cascade in human cardiovascular disease is supported by the CANTOS trial, in which canakinumab reduced recurrent cardiovascular events independently of lipid lowering [99], and by the cardiovascular benefits of colchicine demonstrated in the COLCOT and LoDoCo2 trials [134, 161].

In parallel, vascular microcalcifications arise through osteogenic transdifferentiation of VSMCs, apoptotic body–derived matrix vesicle release, and hydroxyapatite nucleation on a collagen scaffold [27, 32, 118]. Microcalcifications can coalesce into macrocalcifications; paradoxically, dense macrocalcifications may stabilize plaque, whereas small microcalcifications embedded within fibrous caps concentrate mechanical stress and increase rupture risk [32, 119].

### Plaque phenotypes, rupture, and clinical consequences

Plaque structural composition and inflammatory activity determine clinical behavior [162, 163]. Stable plaques harbor thick fibrous caps with abundant VSMCs and collagen, small necrotic cores, and fewer inflammatory cells; they cause gradual luminal narrowing and stable angina. Vulnerable plaques exhibit thin, collagen-poor caps, large necrotic cores, and dense macrophage and T-cell infiltration; activated macrophages secrete matrix metalloproteinases that degrade cap collagen, rendering plaques susceptible to rupture and thrombosis, causing acute myocardial infarction and ischemic stroke [162, 163]. Superficial erosion, associated with neutrophil extracellular traps and endothelial injury without frank plaque rupture, represents an additional, increasingly recognized thrombotic mechanism [162].

In summary, atherosclerosis illustrates the complete deposition disease spectrum: from initiating lipoprotein retention driven by ECM proteoglycans [138, 142, 147], through foam cell formation and impaired RCT [62, 64, 149], defective efferocytosis and necrotic core expansion [98, 160], crystalline DAMP-driven NLRP3 activation [12, 50, 164], and mineral deposition [27, 32, 118], to clinical events driven by plaque instability [162, 163]. Each of these steps has mechanistic parallels in other deposition diseases, as the following sections demonstrate.

## Alzheimer's disease: a protein deposition disorder with a lipid dimension

If atherosclerosis is the vascular archetype of deposition, Alzheimer’s disease is its neurodegenerative counterpart: a disorder in which abnormal production, structural conversion, failed clearance, and chronic innate immune activation transform soluble proteins into persistent and toxic tissue deposits. Importantly, AD is not solely a protein aggregation disease in the narrow sense, because the accumulation, clearance, and inflammatory consequences of Aβ and tau are deeply shaped by lipid handling, vascular function, and glial metabolism. The following subsections therefore treat AD not as an isolated proteinopathy, but as a deposition disorder that shares core biological logic with atherosclerosis while preserving its own disease-specific anatomy and molecular triggers.

### APP processing, Aβ generation, and aggregation

AD neuropathology is defined by extracellular Aβ plaques together with intraneuronal and/or extracellular tau neurofibrillary tangles [10, 11, 15–17]. Aβ itself is not deposited as a native cellular product; rather, it is generated from the amyloid precursor protein (APP), a type-I transmembrane glycoprotein whose physiological functions include synaptic plasticity modulation and iron export [11, 16, 120]. In the non-amyloidogenic pathway, APP is cleaved by α-secretase within the Aβ domain, precluding amyloid formation. In the amyloidogenic pathway, APP is cleaved by BACE1 (β-secretase), the transmembrane aspartic protease whose identification as the neuronal β-secretase established the molecular basis of amyloidogenic APP processing [121], to yield a membrane-bound C-terminal fragment (C99), which is subsequently cleaved by the presenilin-1– or presenilin-2–containing γ-secretase complex, generating Aβ peptides of 37–43 amino acids [11, 16, 120–123]. The catalytic mechanism of γ-secretase depends on two transmembrane aspartate residues within presenilin that form the active site of this intramembrane protease [122]. Familial AD mutations in APP, PSEN1, or PSEN2 shift processing toward the longer, β-sheet–prone Aβ42 species, which aggregates more rapidly than Aβ40 [16, 122, 123]. Sporadic AD, which accounts for > 95% of cases, is driven by impaired Aβ clearance in the context of aging and genetic risk, most notably the APOE ε4 allele [59].

Aβ monomers assemble hierarchically into soluble oligomers, protofibrils, and insoluble amyloid fibrils that deposit as neuritic and diffuse plaques [15, 17, 75]. Soluble oligomeric Aβ species, particularly dimers and low-molecular-weight oligomers isolated from AD brain tissue, are acutely synaptotoxic, impairing long-term potentiation, disrupting glutamate receptor trafficking, and inducing dendritic spine loss [17, 165].

### Aβ clearance: proteolytic, vascular, and glymphatic mechanisms

Aβ levels reflect a dynamic balance between production and clearance. Major clearance routes include: proteolytic degradation by neprilysin, insulin-degrading enzyme (IDE), matrix metalloproteinases, and plasmin [47, 49]; BBB transport mediated by LRP1 (efflux from brain) and RAGE (influx into brain) [47, 49]; perivascular and glymphatic efflux pathways that drain interstitial solutes along paravascular spaces to the CSF and cervical lymphatics [48, 51]; and microglial phagocytic clearance [124–128]. It is important to note all four clearance mechanisms at this stage, as their collective impairment, rather than any single pathway's failure, underlies the production–clearance imbalance that drives AD. The glymphatic system, driven by aquaporin-4 (AQP4)–facilitated convective flow, is particularly active during sleep; sleep disruption in aging impairs glymphatic function and accelerates Aβ accumulation [130]. Glymphatic failure has been proposed as a final common pathway to dementia in multiple neurodegenerative conditions [129]. Aging, vascular pathology, and APOE ε4 collectively impair all these clearance routes [47–49, 51, 59].

### Tau pathology, propagation, and neurofibrillary tangles

Tau is a microtubule-associated protein encoded by the MAPT gene, expressed predominantly in axons where it stabilizes microtubule polymerization [15, 166]. In AD and other tauopathies, abnormal hyperphosphorylation at multiple serine and threonine residues causes tau to detach from microtubules, disrupt axonal transport, and aggregate into paired helical filaments (PHFs) that form neurofibrillary tangles (NFTs) [15, 166]. The stereotyped anatomical progression of tau pathology through Braak stages, from transentorhinal cortex to limbic structures to neocortex, correlates more closely with the degree of cognitive impairment than amyloid plaque burden does [167]. Experimental and neuropathological evidence supports “prion-like” templated propagation of tau aggregates along synaptically connected networks [166–169]. The synergistic relationship between Aβ and tau, whereby amyloid pathology promotes tau propagation and their combined neurotoxicity exceeds additive effects, represents a critical axis of AD pathobiology [170].

### Glial responses, neuroinflammation, and the NLRP3 inflammasome

Aβ and tau deposits elicit robust glial responses that are integral to, rather than merely secondary to, AD pathogenesis [125, 126, 128, 171]. Microglia, the brain-resident innate immune cells, recognize Aβ via TREM2, TLRs (TLR2, TLR4), complement receptors, and scavenger receptors; initially, microglia cluster around plaques and mediate phagocytic clearance [124–128]. The microglial sensory apparatus, or “sensome,” enabling recognition of Aβ, lipoproteins, and other DAMPs has been comprehensively characterized by transcriptomic approaches [127]. The barrier function of plaque-associated microglia in restricting diffusion of neurotoxic Aβ species has been directly demonstrated [124]. Disease-associated microglia (DAM), identified by single-cell transcriptomics, upregulate lipid metabolism and phagocytic genes while downregulating homeostatic signatures; their ability to clear Aβ diminishes with chronic plaque burden and aging [125, 128]. TREM2 signaling regulates microglial cholesterol metabolism upon chronic phagocytic challenge, linking TREM2-mediated phagocytosis directly to the lipid dimension of AD [172]. Genome-wide association studies implicating microglial and immune-related genes, including TREM2, CD33, CR1, CLU, BIN1, and ABCA7, underscore the fundamental importance of innate immunity–deposition interactions in AD [125, 173, 174].

There is now substantial evidence that the NLRP3 inflammasome plays a central role in AD pathogenesis, paralleling its role in atherosclerosis and gout, a convergence that directly supports the unified deposition framework of this review [101, 102, 146, 175]. Aβ fibrils and oligomers activate the NLRP3 inflammasome in microglia, driving caspase-1 activation and IL-1β/IL-18 production; genetic or pharmacological inhibition of NLRP3 reduces amyloid burden and rescues cognitive deficits in APP/PS1 mice [101]. Microglia-derived ASC specks, protein complexes that form during inflammasome activation, can cross-seed Aβ aggregation, providing a direct mechanistic link between neuroinflammation and plaque growth [146]. Critically, NLRP3 activation also drives tau pathology: NLRP3-deficient tau transgenic mice display attenuated NFT formation and neurodegeneration, and the NLRP3/ASC axis modulates tau pathology in tauopathy models [102, 175]. This positions NLRP3 inflammasome activation as a convergent mechanistic node shared by atherosclerosis (Sect. " Cholesterol crystals, NLRP3 inflammasome activation, and calcification"), AD, and related deposition diseases (Sect. " Innate immunity, pattern recognition, and NLRP3 inflammasome activation"), and as an attractive cross-disease therapeutic target [101, 102, 146, 175, 176].

Astrocytes become reactive (astrogliosis) in the vicinity of plaques and tangles, contributing to gliosis, disrupted glutamate homeostasis, synaptic remodeling, and sustained inflammatory mediator release [171]. Complement activation by Aβ deposits promotes further microglial recruitment and synaptic pruning by complement-tagged synapses [125, 174].

### Lipid dysregulation in Alzheimer's disease

While AD has traditionally been categorized as a protein deposition disorder, emerging evidence reveals substantial lipid dyshomeostasis as a co-pathological feature that fundamentally challenges this categorization and reinforces the convergence with atherosclerosis [67, 71]. Comprehensive lipidomic analysis of AD brain tissue reveals widespread alterations in sphingolipids, phospholipids, cholesterol esters, and glycerophospholipids, with disruptions in membrane lipid composition affecting Aβ processing, tau phosphorylation, and synaptic function [71]. The molecular mechanisms include cholesterol-dependent modulation of APP processing, increased cholesterol promotes amyloidogenic cleavage by facilitating BACE1 and γ-secretase activity in lipid raft microdomains, altered ganglioside composition facilitating Aβ nucleation on neuronal membranes, and ceramide-mediated promotion of tau hyperphosphorylation [71, 177].

Lipid-droplet-accumulating microglia (LDAM) represent a dysfunctional, pro-inflammatory microglial state in the aging brain and in AD [67]. LDAMs accumulate neutral lipids, triglycerides and cholesterol esters, in cytoplasmic lipid droplets, a state associated with reduced phagocytic capacity, increased ROS production, and a pro-inflammatory transcriptional profile [67]. The striking parallel between LDAMs in the aging brain and lipid-laden foam cell macrophages in atherosclerotic plaques (Sect. " Lipoprotein modification and foam cell formation") reinforces the conceptual convergence between vascular and neurodegenerative deposition diseases at a cellular level [67]. TREM2 signaling regulates microglial cholesterol metabolism and influences the transition toward lipid-laden dysfunctional states [172]. APOE ε4 impairs lipid transport and cholesterol efflux in the brain, further integrating lipid metabolism into AD pathogenesis [59, 173].

### Cerebral amyloid angiopathy

Aβ, predominantly the Aβ40 isoform, also deposits in the walls of leptomeningeal and cortical arteries and arterioles, causing cerebral amyloid angiopathy (CAA) [52, 178]. CAA is a leading cause of lobar intracerebral hemorrhage and cerebral microbleeds and is present to some degree in more than 80% of AD patients [52, 178]. Vascular Aβ accumulation compromises vessel wall integrity, impairs smooth muscle cell function and vasoreactivity, and critically undermines perivascular clearance of interstitial solutes, creating a vicious cycle that promotes further parenchymal Aβ deposition [51, 52]. CAA exemplifies the vascular manifestation of protein deposition and represents a direct anatomical bridge between the vascular and neurodegenerative deposition paradigms described in this review.

**Fig. 4:**
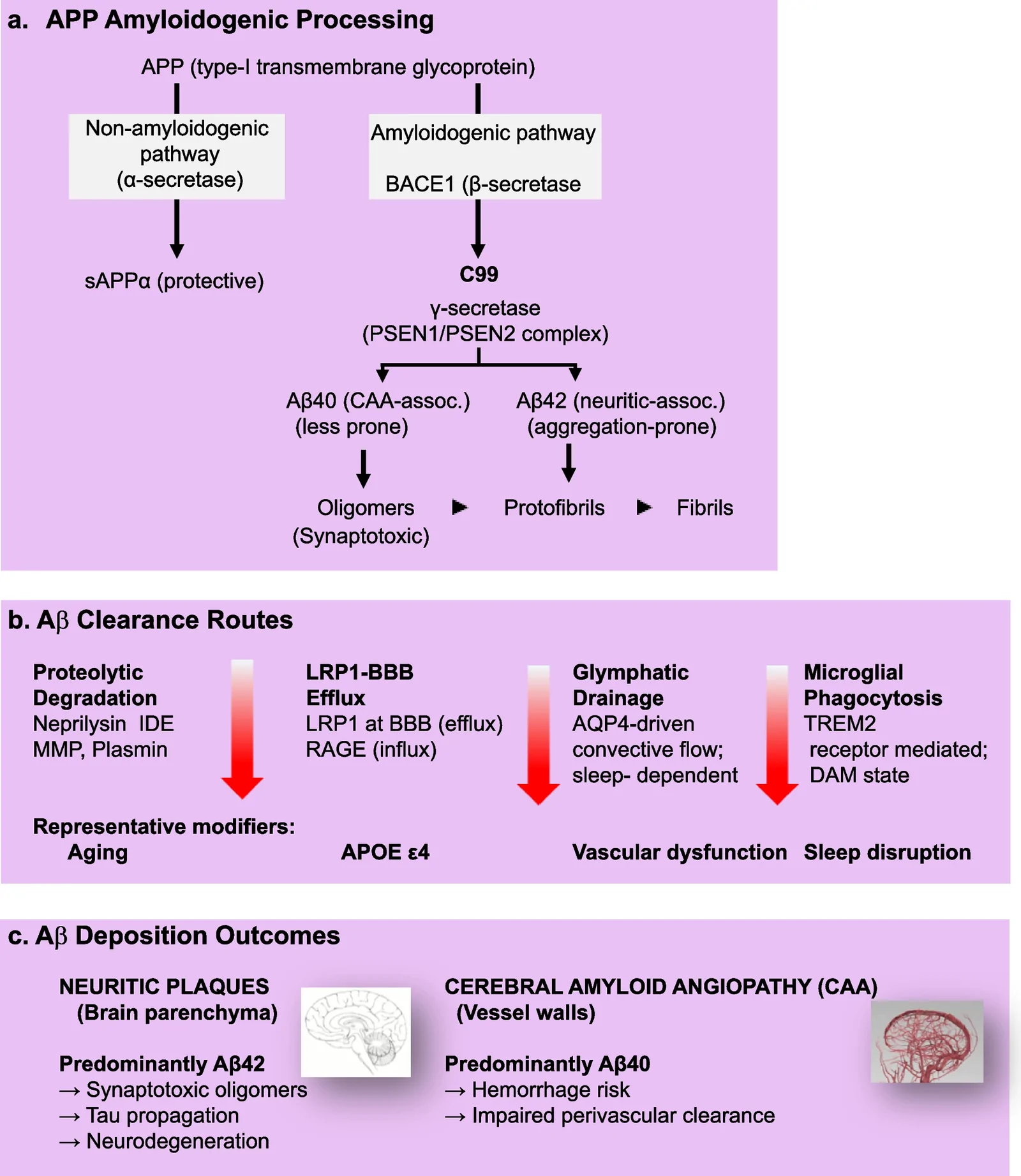
Mechanisms of Aβ generation, clearance, and deposition in Alzheimer’s disease. Integrated schematic of amyloid-β (Aβ) generation, clearance, and deposition in Alzheimer’s disease. **a** Amyloid precursor protein (APP), a type-I transmembrane glycoprotein, undergoes non-amyloidogenic processing via α-secretase or amyloidogenic processing via BACE1 (β-secretase) followed by γ-secretase (presenilin-1/2 complex), generating Aβ40 and Aβ42. Soluble Aβ species assemble into oligomers, protofibrils, and fibrils, with oligomeric intermediates exerting synaptotoxicity. **b** Major Aβ clearance pathways include proteolytic degradation by neprilysin and insulin-degrading enzyme, blood–brain barrier efflux mediated by LRP1, glymphatic drainage driven by aquaporin-4 (AQP4), and TREM2-dependent microglial phagocytosis. Red gradient arrows indicate progressive impairment of these pathways, and the labels below identify representative modifiers, including aging, APOE ε4, vascular dysfunction, and sleep disruption. **c** Clearance failure promotes deposition of Aβ in brain parenchyma as neuritic plaques, composed predominantly of Aβ42, and in cerebral vessel walls as cerebral amyloid angiopathy (CAA), composed predominantly of Aβ40. These deposition patterns contribute to synaptotoxicity, tau propagation, neurodegeneration, vascular fragility, and impaired perivascular solute clearance

Taken together, AD demonstrates that deposition pathology in the brain is not simply a matter of protein aggregation, but the outcome of a tightly interwoven system involving precursor processing, failed proteolytic and vascular clearance, glial dysfunction, lipid dyshomeostasis, and self-amplifying neuroinflammation. The integrated model of Aβ generation and clearance is shown in Fig. 4. This broader interpretation of AD is important because it allows the disease to be placed on the same mechanistic continuum as non-neurological deposition disorders. The disorders discussed next, systemic amyloidoses, immune complex diseases, crystal arthropathies, and lysosomal storage disorders differ in substrate and tissue distribution, but they recapitulate many of the same pathogenic motifs.

## Other major deposition disorders

The diseases described in this section extend the deposition framework beyond the two primary paradigms. Rather than cataloging these disorders independently, this section emphasizes how each illuminates a particular aspect of the shared pathophysiology and connects back mechanistically to atherosclerosis and AD. A key organizing theme is that the downstream inflammatory effector response, whether triggered by lipids, protein fibrils, crystals, or immune complexes, consistently converges on NLRP3 activation, complement engagement, and cytokine-driven tissue injury [17].

### Systemic amyloidoses

Systemic amyloidoses are characterized by extracellular deposition of misfolded proteins as amyloid fibrils in multiple organs [17, 28, 30, 35, 38, 75]. Despite heterogeneous precursor proteins, all amyloid fibrils share a cross-β-sheet architecture, bind Congo red with apple-green birefringence under polarized light, fluoresce with Thioflavin T/S, and associate with ECM components including heparan sulfate proteoglycans and serum amyloid P component [75, 140, 141].

In AL amyloidosis, monoclonal immunoglobulin light chains produced by bone marrow plasma cell clones misfold and aggregate, most commonly affecting the heart, kidneys, liver, and peripheral/autonomic nerves [28, 36]. AL cardiac amyloidosis diagnosis and therapy continue to evolve, with daratumumab-based regimens achieving deep hematological responses and organ improvement [28]. ATTR amyloidosis arises from misfolding of transthyretin; hereditary ATTR is caused by TTR mutations (e.g., Val122Ile, Val30Met), while wild-type ATTR primarily affects the aging heart and carpal tunnel [30, 35, 107]. AA amyloidosis occurs in chronic inflammatory states with sustained SAA production [38].

ApoA-I amyloidosis represents a uniquely instructive case in which the same protein that mediates atheroprotective RCT, when mutated or post-translationally modified, becomes an amyloidogenic precursor—directly connecting the lipid and protein deposition categories [61–64]. Organ injury in systemic amyloidoses results from mechanical disruption, microvascular compromise, and direct cytotoxicity of prefibrillar oligomeric and fibrillar amyloid species [30, 35, 36, 38].

### Immune complex–mediated deposition

Immune complex deposition underlies a mechanistically distinct but thematically convergent class of disorders [25, 33, 40, 42, 112, 179]. In lupus nephritis (LN), nucleic acid–protein immune complexes deposit in glomerular capillary walls and mesangium, activate the classical complement pathway, and recruit neutrophils and monocytes, culminating in proliferative and sclerosing lesions [25, 42]. Membranous nephropathy involves subepithelial immune complex formation against podocyte antigens (most commonly PLA2R), activating complement and causing nephrotic syndrome [40]. Immune complex vasculitis features immune complex deposition in vessel walls, complement activation, and leukocytoclastic inflammation [33, 114].

The conceptual connection to the primary paradigms is mechanistically grounded: deposition in vessel walls occurs in both atherosclerosis (subendothelial lipid deposits) and immune complex vasculitis (immune complex deposits in vessel walls), with both activating downstream inflammatory cascades [33, 34, 50]; complement activation is prominent in both lupus nephritis and in Aβ-mediated complement-driven synaptic pruning in AD [125, 174], indicating that the complement system is a shared effector of tissue injury regardless of the nature of the triggering deposit; and leukocytoclastic inflammation in immune complex vasculitis shares neutrophil-driven mechanisms with sterile inflammation in crystal arthropathies and atherosclerosis [33, 34, 50]. These parallels reinforce the conceptual unity of deposition-driven inflammation and strengthen the case for shared therapeutic strategies [112, 179].

### Crystal deposition diseases

Crystalline deposits are among the most potent activators of innate immunity, and their pathophysiology most directly illustrates the convergent role of NLRP3 inflammasome activation across deposition diseases [26, 34, 41, 50, 111]. In gout, monosodium urate (MSU) crystals precipitate in joints and periarticular tissues under conditions of hyperuricemia. MSU crystals are phagocytosed by synovial macrophages and neutrophils; the crystals destabilize the phagolysosomal membrane and activate the NLRP3 inflammasome, resulting in massive IL-1β release and the exquisitely painful, self-limited acute gouty attack [26, 34, 50, 111]. Animal model evidence has established the NLRP3 inflammasome as the critical driver of sterile crystal-induced inflammatory disease [111]. Calcium pyrophosphate deposition disease (CPPD) involves CPPD crystal deposition in articular cartilage and synovium with similar NLRP3-mediated inflammation [41].

The thematic unity of NLRP3 inflammasome activation by cholesterol crystals in atherosclerosis [12, 50], Aβ in AD [101, 146], and urate or CPPD crystals in arthropathies [26, 50, 111] reflects the NLRP3 system's conserved role as a sensor of structurally dangerous, crystalline, fibrillar, or particulate, endogenous materials. This convergence, detailed mechanistically in Sect. " Innate immunity, pattern recognition, and NLRP3 inflammasome activation", positions NLRP3 as the single most compelling cross-disease therapeutic target identified in this review [176].

### Metabolic storage and lysosomal disorders

Lysosomal storage diseases (LSDs) arise from inherited defects in lysosomal hydrolases, transport proteins, or cofactors, resulting in progressive accumulation of undegraded substrates within lysosomes [29, 31, 37, 39, 91]. Gaucher disease results in glucocerebroside-laden macrophages in spleen, liver, bone marrow, and, in neuronopathic forms, the CNS [31, 116]. Heterozygous GBA1 mutations are the most common genetic risk factor for Parkinson's disease, directly linking lysosomal dysfunction to α-synuclein aggregation and synucleinopathy [115]. Fabry disease is characterized by Gb3 accumulation in vascular endothelium, podocytes, cardiomyocytes, and peripheral neurons [29]. Niemann-Pick disease type C (NPC1/NPC2 mutations) causes cholesterol and sphingolipid trafficking defects; type C disease is notable for its connection to tau pathology and cholesterol dyshomeostasis, with established diagnostic and management guidelines [117]. Mucopolysaccharidoses involve glycosaminoglycan storage with multisystem involvement [37].

LSDs differ from atherosclerosis, AD, and systemic amyloidoses in that deposition is predominantly intracellular and results from impaired catabolism. However, this is the critical point of mechanistic convergence as they share downstream consequences that connect them to extracellular deposition diseases: lysosomal membrane permeabilization and release of cathepsins activates NLRP3 [39, 50, 115, 145]; mitochondrial dysfunction and ER stress are shared with AD neurons [39, 131–133]; and neuroinflammation driven by lipid-laden macrophages in Gaucher disease parallels foam cell inflammation in atherosclerosis and LDAM neuroinflammation in AD [31, 115, 116]. Furthermore, the lysosomal dysfunction central to LSDs resonates directly with the impaired autophagic and lysosomal clearance of Aβ and tau in AD [131–133], placing both on a continuum of defective intracellular protein and lipid homeostasis. These convergent secondary pathogenic cascades (oxidative stress, calcium dysregulation, and neuroinflammation) are shared across multiple LSDs despite heterogeneous primary enzyme defects [135].

## Shared molecular pathways across deposition diseases

Having compared individual deposition diseases, the next step is to identify the molecular systems that repeatedly appear across them. Three such systems recur with striking consistency: proteostasis and clearance networks, innate immune sensing and inflammasome activation, and the extracellular matrix/tissue microenvironment. These are not parallel topics but interacting layers of a single pathogenic architecture, together determining whether deposited material is eliminated, stabilized, or converted into a chronic inflammatory nidus.

### Proteostasis and clearance mechanisms

Impaired proteostasis, the collective system maintaining protein synthesis, folding, and degradation in balance, is central to protein deposition diseases [131–133]. The ubiquitin–proteasome system (UPS) mediates degradation of short-lived and misfolded cytosolic proteins; proteasome dysfunction is observed in AD neurons, α-synucleinopathies, and some systemic amyloidoses [131, 133]. The autophagy-lysosome pathway handles long-lived proteins, protein aggregates, and damaged organelles; impaired macroautophagy and chaperone-mediated autophagy (CMA) contribute to aggregate accumulation in AD and Parkinson's disease [131, 132]. The unfolded protein response (UPR) of the ER provides a third line of defense; chronic ER stress resulting from persistent misfolded protein load activates pro-apoptotic UPR branches and contributes to neuronal loss in AD [132, 180].

Phagocytic clearance is the critical extracellular counterpart to intracellular proteostasis. Microglial phagocytosis of Aβ, mediated by TREM2, complement receptors, scavenger receptors, and LRP1, represents a key Aβ clearance mechanism whose efficiency declines with aging and APOE ε4 burden [124, 125, 127, 128, 145, 172]. Macrophage efferocytosis of apoptotic foam cells in atherosclerotic plaques is comparably important for preventing necrotic core expansion [98]. The mechanistic parallel between these two phagocytic clearance systems, both dependent on scavenger receptor signaling, both impaired by the same inflammatory microenvironment, and both targetable by similar receptor-modulating strategies, is one of the most compelling synergies identified in this review, discussed further in Sect. " Enhancing clearance: cross-disease synergies". Fluid clearance via glymphatic and lymphatic pathways removes extracellular Aβ and other solutes from the CNS; age-related AQP4 mislocalization, sleep disruption, and vascular stiffening impair glymphatic clearance and are emerging therapeutic targets [48, 51, 129, 130].

### Innate immunity, pattern recognition, and NLRP3 inflammasome activation

Deposited materials, whether oxidized lipoproteins, amyloid fibrils, crystals, or immune complexes, converge on innate immune pathways by acting as DAMPs that engage PRRs [50, 99, 144, 145]. TLR4 and TLR2 recognize oxLDL and Aβ fibrils, activating NF-κB and inducing pro-inflammatory gene expression in macrophages and microglia [128, 144]. Scavenger receptors (SR-A, CD36, MARCO) mediate both pro-inflammatory signaling and phagocytic clearance in a context-dependent manner [136, 137, 148].

**Fig. 5:**
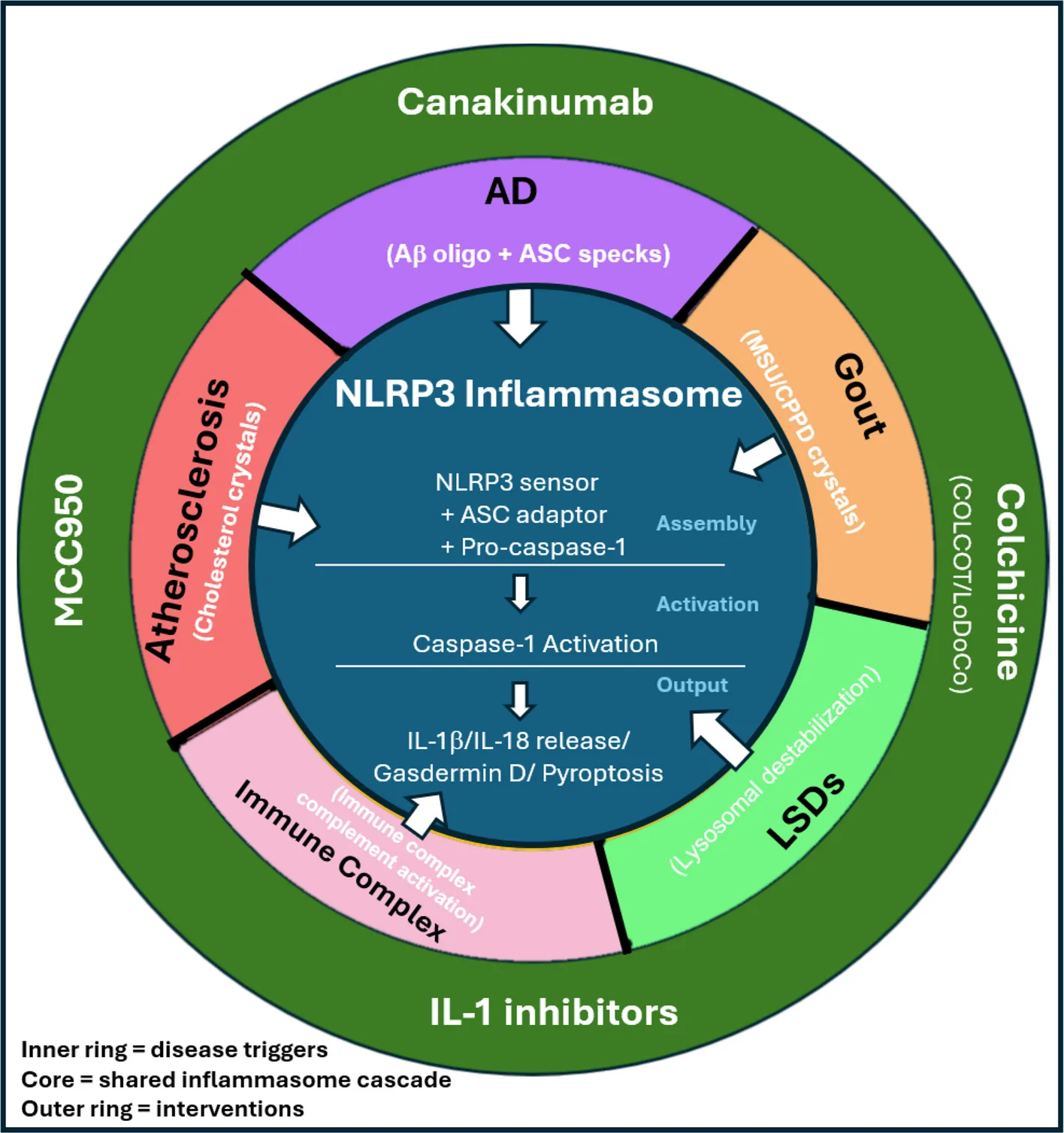
NLRP3 inflammasome activation as a convergent inflammatory node across deposition diseases. Radial schematic illustrating NLRP3 inflammasome activation as a shared inflammatory convergence point across multiple deposition disorders. The inner ring represents disease-specific triggers, including cholesterol crystals in atherosclerosis, Aβ oligomers and ASC specks in Alzheimer’s disease, monosodium urate (MSU) and calcium pyrophosphate dihydrate (CPPD) crystals in crystal arthropathies, lysosomal destabilization in lysosomal storage disorders (LSDs), and immune complex/complement-associated signals in immune complex diseases. These triggers converge on a common central inflammasome cascade comprising assembly of the NLRP3–ASC–pro-caspase-1 complex, activation of caspase-1, and output in the form of IL-1β/IL-18 release and gasdermin D–mediated pyroptosis. The outer ring denotes representative interventions targeting this pathway, including canakinumab, colchicine, IL-1 inhibitors, and the preclinical NLRP3 inhibitor MCC950. The labels “inner ring,” “core,” and “outer ring” identify the three conceptual levels of the figure: disease triggers, shared inflammasome cascade, and therapeutic intervention, respectively

The NLRP3 inflammasome, comprising the sensor NLRP3, the adaptor ASC, and the effector caspase-1, is the prototypical innate immune amplifier in deposition diseases and the convergent node most consistently activated across disease categories [50, 145]. NLRP3 is activated by cholesterol crystals [12, 50], MSU crystals [34, 50, 111], Aβ oligomers and fibrils [101, 146], lysosomal damage, potassium efflux, and mitochondrial ROS [50, 145]. Upon activation, caspase-1 cleaves pro-IL-1β and pro-IL-18 to their active forms and induces gasdermin D–mediated pyroptotic cell death [145]. The clinical relevance of this pathway is demonstrated by the CANTOS trial [99] and by the cardiovascular benefits of colchicine [134, 161], while preclinical evidence demonstrates that NLRP3 inhibition reduces amyloid burden and tau pathology in AD models [101, 102, 175]. The mechanistic connections between NLRP3-driven inflammation and the broader inflammatory hypothesis of atherosclerosis have been comprehensively reviewed [176]. Cross-disease inflammasome convergence is summarized in Fig. 5.

### Extracellular matrix and tissue microenvironment

The extracellular matrix and local milieu profoundly influence the propensity for, and stability of, pathological deposits [138–143]. Arterial proteoglycans, including biglycan, decorin, and versican, bind apoB-100 via electrostatic interactions, retaining LDL in the subendothelial space and facilitating its oxidative modification [138, 142, 147]. Heparan sulfate proteoglycans are universally enriched in systemic amyloid deposits, including AA, AL, ATTR, and Aβ, where they lower the activation energy for fibrillogenesis and protect fibrils from proteolytic degradation [140, 141]. Pharmacological targeting of HSPG–amyloid interactions has been explored as a therapeutic strategy [139]. Collagen and fibronectin matrices provide scaffolds for mineral nucleation in vascular calcification, with lysine post-translational modifications of collagen determining scaffold properties and hydroxyapatite binding efficiency [27, 33, 143].

Local physicochemical conditions further modulate deposition: arterial pH gradients and oxidative microenvironments favor LDL oxidation and cholesterol crystallization [136, 137]; low pH in joints facilitates urate crystal stability [26]; and reduced brain interstitial pH in hypoxia and aging may modulate Aβ aggregation kinetics [75]. Tissue-specific ECM composition helps explain the characteristic organ tropism of each deposit and represents a microenvironmental axis for therapeutic targeting across disease categories [138, 181].

## Diagnostic approaches

A synthesis-driven view of deposition disease has diagnostic implications: if these disorders share common biological architectures, then their assessment should likewise be understood in terms of three recurring questions, what is deposited, where it is deposited, and how actively the deposit is driving tissue injury. Histopathology, molecular imaging, and fluid biomarkers address these questions in different ways across organ systems. The value of reviewing diagnostics here is therefore not simply practical, but conceptual: it shows how diverse diseases can be measured using analogous principles of burden, localization, and biological activity.

### Histopathology and special stains

Classical histopathology remains the gold standard for definitive diagnosis. In atherosclerosis, H&E staining reveals intimal thickening, foam cell accumulation, necrotic cores, and fibrous caps; Oil Red O or Sudan IV on frozen sections identifies neutral lipids; von Kossa and Alizarin red stains highlight calcification; immunostaining for CD68 and α-SMA characterizes cellular composition [5, 6, 9]. Amyloid deposits are detected by Congo red with apple-green birefringence under polarized light, Thioflavin T/S fluorescence microscopy, and precursor-specific immunohistochemistry (Aβ, TTR, κ/λ light chains, SAA) [35, 36, 38, 75]. Neurodegenerative proteinopathies require immunostaining for Aβ, phosphorylated tau (AT8, PHF-1 epitopes), α-synuclein, and TDP-43 [11, 15, 17, 79, 128]. Immune complex diseases are assessed by direct immunofluorescence microscopy for immunoglobulin classes and complement components in renal biopsies [25, 33, 40, 42]. Crystals are identified by compensated polarized light microscopy: MSU crystals display strong negative birefringence; CPPD crystals show weak positive birefringence [26, 34, 41].

### In vivo imaging

Non-invasive imaging has transformed clinical assessment and monitoring of deposition diseases [182]. In atherosclerosis, carotid B-mode ultrasound quantifies intima-media thickness [183]; coronary CT angiography characterizes plaque morphology and calcium burden via Agatston scoring [184]; 18F-NaF PET identifies active microcalcification; and 18F-FDG PET-CT maps plaque inflammation [182, 185]. In AD, structural MRI detects region-specific atrophy; 11C-PiB and 18F-florbetapir/flutemetamol PET quantify fibrillar Aβ burden [186, 187]; and 18F-flortaucipir PET images NFT distribution in accordance with Braak staging [187]. The NIA-AA Research Framework integrates these imaging modalities into a biological definition of AD based on ATN (Amyloid, Tau, Neurodegeneration) biomarkers [188]. Technetium-99 m–labeled bone-seeking tracers enable non-biopsy diagnosis of ATTR cardiac amyloidosis [189].

### Fluid and molecular biomarkers

Plasma and CSF biomarkers enable disease characterization and longitudinal monitoring. In atherosclerosis, LDL-C, non-HDL-C, Lp(a), and apoB quantify atherogenic lipoprotein burden; hsCRP reflects systemic inflammation; and emerging biomarkers include oxidized phospholipids and soluble PCSK9 [5, 6, 9, 13, 190]. In AD, the established CSF biomarker triplet, Aβ42/Aβ40 ratio, total tau (t-tau), and phosphorylated tau-181 (p-tau181), provides high diagnostic accuracy [188, 191, 192]; plasma p-tau181, p-tau217, p-tau231, Aβ42/Aβ40 ratio, and neurofilament light chain (NfL) are validated blood-based biomarkers now entering clinical use [192–194]. In systemic amyloidosis, serum free light chains and immunofixation electrophoresis assess AL disease; TTR genotyping distinguishes hereditary from wild-type ATTR; and SAA levels guide AA amyloidosis monitoring [35, 36, 38]. Biomarker integration across modalities increasingly supports precision diagnosis and treatment selection.

## Therapeutic strategies targeting deposition biology

**Fig. 6:**
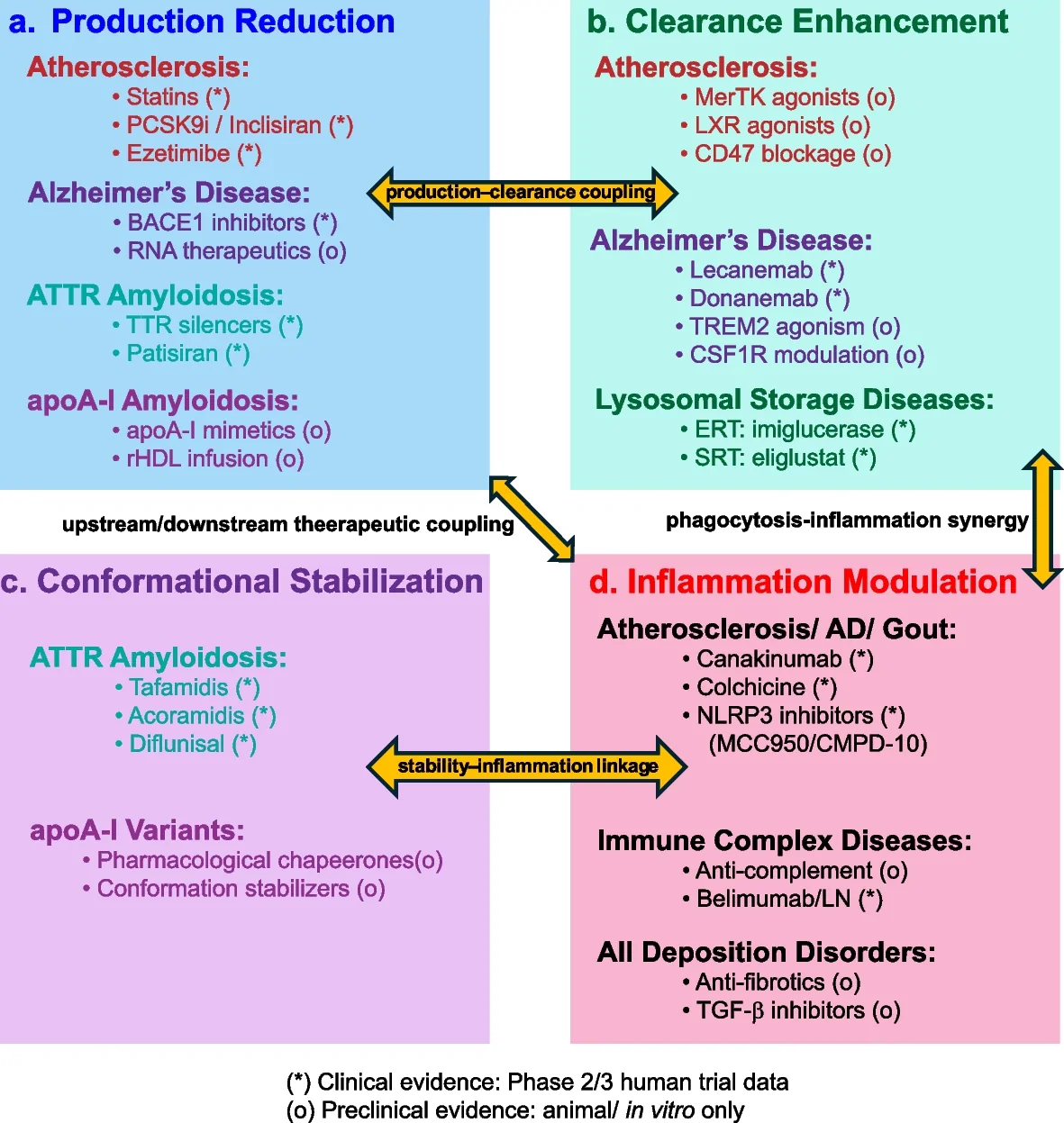
Therapeutic landscape for deposition diseases organized by mechanistic category and cross-disease synergies. Four-quadrant schematic summarizing therapeutic strategies across deposition diseases according to mechanism of action. **a** Production reduction includes therapies that decrease generation or systemic burden of deposited material, such as statins, PCSK9 inhibitors/inclisiran, and ezetimibe in atherosclerosis; BACE1 inhibitors and RNA therapeutics in Alzheimer’s disease; TTR silencers and patisiran in ATTR amyloidosis; and apoA-I mimetics or recombinant HDL approaches in apoA-I dysfunction. **b** Clearance enhancement includes therapies that augment removal of deposited material, including lecanemab and donanemab in Alzheimer’s disease, TREM2 agonism and CSF1R modulation in microglial clearance, MerTK agonists, LXR agonists, and CD47 blockade in atherosclerosis, and enzyme replacement therapy (ERT) or substrate reduction therapy (SRT) in lysosomal storage disorders. **c** Conformational stabilization includes therapies that preserve native protein structure and reduce misfolding, including tafamidis, acoramidis, and diflunisal in ATTR amyloidosis, and pharmacological chaperones or other conformation-stabilizing strategies for apoA-I variants. **d** Inflammation modulation includes therapies targeting shared downstream inflammatory pathways, including canakinumab, colchicine, and NLRP3 inhibitors across atherosclerosis, Alzheimer’s disease, and gout; anti-complement strategies in immune complex diseases; and anti-fibrotic/TGF-β–directed approaches across multiple deposition disorders. Labeled bidirectional arrows highlight proposed cross-disease synergies, including production–clearance coupling, upstream/downstream therapeutic coupling, phagocytosis–inflammation synergy, and stability–inflammation linkage. Asterisks denote therapies supported by clinical trial evidence, whereas open-circle annotations denote preclinical or investigational strategies

If deposition diseases share pathogenic logic, then their therapies can also be organized by shared intervention points rather than by organ system alone. Across disorders, treatment strategies repeatedly target one or more of four mechanistic vulnerabilities: reducing production of the deposited material, enhancing its clearance, stabilizing native conformations to prevent misfolding, or interrupting downstream inflammatory and remodeling cascades. Organizing therapy in this way highlights where disease-specific interventions remain necessary, but also where cross-disease repurposing and mechanistic synergies become plausible. The therapeutic landscape is outlined in Fig. 6.

### Reducing pathological production

In atherosclerosis, statins, ezetimibe, and PCSK9 inhibitors reduce hepatic apoB lipoprotein secretion and increase LDL receptor recycling, achieving LDL-C reductions of up to 85% and promoting plaque regression [100, 195–197]. RNA-targeted therapies, inclisiran (siRNA against PCSK9) [198] and obicetrapib [199], expand the toolkit for sustained LDL-C lowering. The REDUCE-IT trial demonstrated cardiovascular benefits of icosapentaenoic acid supplementation [200]. Antisense oligonucleotides targeting Lp(a) and apoC-III address residual lipid-mediated risk [190].

In AD, BACE1 inhibitors reduce Aβ production but were largely unsuccessful in phase III trials due to mechanism-based side effects and, likely, intervention too late in the disease course [201, 202]. In AL amyloidosis, plasma cell clone suppression with proteasome inhibitors and anti-CD38 monoclonal antibodies (daratumumab) reduces light chain supply and can achieve hematological remission [28, 36]. In AA amyloidosis, biologic therapies targeting IL-1 (anakinra), IL-6 (tocilizumab), and TNF reduce SAA production [38, 109].

Strategies targeting apoA-I–mediated reverse cholesterol transport are under active investigation as both anti-atherosclerotic and potentially anti-amyloid approaches. ApoA-I mimetic peptides (e.g., D-4F), recombinant HDL (rHDL) infusion, and ABCA1 agonists aim to restore cholesterol efflux from macrophage foam cells; some preclinical data additionally suggest that apoA-I can solubilize and facilitate Aβ clearance, a cross-disease synergy that warrants prioritized investigation [62, 64].

### Enhancing clearance: cross-disease synergies

The parallel impairment of phagocytic clearance in both atherosclerosis (defective macrophage efferocytosis) and AD (declining microglial phagocytosis) represents one of the most compelling cross-disease therapeutic opportunities identified in this review. Both systems depend on similar molecular machinery: scavenger receptor signaling, phosphatidylserine recognition, lipid metabolism via ABCA1/ABCG1, and TREM receptor activation [98, 124, 125, 128, 145, 160, 172]. Both are undermined by the same inflammatory microenvironment, and both are being targeted by analogous strategies.

In atherosclerosis, MerTK agonists, LXR agonists promoting ABCA1/ABCG1 expression, and CD47 blockade show preclinical promise for reducing necrotic core expansion [98, 160]. In AD, restoring microglial phagocytic capacity via TREM2 agonism, CSF1R modulation, or APOE4 → APOE3 conversion strategies reduce Aβ burden and neuroinflammation in AD models [124, 125, 128, 145, 172]. The mechanistic overlap is substantial enough to suggest that insights from each field should be systematically mined for application in the other.

Immunotherapy to enhance clearance of pathological protein deposits has achieved important milestones in AD. Lecanemab reduced brain amyloid burden by approximately 50% and slowed cognitive decline by 27% over 18 months in early AD in the CLARITY AD trial [104]. Donanemab achieved robust amyloid clearance and slowed decline in individuals with low-to-medium tau burden in the TRAILBLAZER-ALZ 2 trial [103]. Both agents cause ARIA, particularly in APOE ε4 carriers, requiring MRI monitoring [103, 104, 203]. The 2024 AD drug development pipeline documents the expanding portfolio of anti-Aβ, anti-tau, and anti-neuroinflammation programs [204].

In lysosomal storage diseases, enzyme replacement therapy and substrate reduction therapy provide substrate clearance and improve clinical outcomes [29, 31, 205].

### Stabilizing native protein conformations

For ATTR amyloidosis, small-molecule TTR kinetic stabilizers (tafamidis and diflunisal) thermodynamically stabilize the TTR tetramer against dissociation into amyloidogenic monomers. Tafamidis demonstrated a 30% reduction in all-cause mortality in the ATTR-ACT trial [35, 106]. RNA-targeted agents (patisiran, vutrisiran, eplontersen) silence hepatic TTR production, achieving > 80% reduction in serum TTR [108, 206]. Small-molecule stabilizers and pharmacological chaperones are being explored for other amyloidogenic proteins including apoA-I variants [63, 101].

### Modulating inflammation and remodeling: NLRP3 as a cross-disease target

The convergent role of NLRP3 inflammasome activation across atherosclerosis, AD, gout, and lysosomal disorders raises the prospect of NLRP3 inhibition as a genuinely cross-disease therapeutic strategy [99, 101, 102, 134, 161, 164, 175, 176]. The landmark CANTOS trial demonstrated that canakinumab (anti-IL-1β) reduced recurrent major adverse cardiovascular events independently of lipid lowering [99]. Post-hoc CANTOS analyses revealed pleiotropic anti-inflammatory effects including a reduction in incident lung cancer [164]. Colchicine demonstrated cardiovascular benefit in the COLCOT trial (post-myocardial infarction) [161] and LoDoCo2 trial (stable coronary disease) [134]. In gout, IL-1β inhibitors effectively terminate acute attacks and prevent recurrence [34]. In AD, NLRP3 inhibitor development (e.g., MCC950/CMPD-10) has shown preclinical efficacy in reducing amyloid burden and improving cognition [101, 102, 175], strongly supporting clinical translation. This translational opportunity, using insights from cardiovascular NLRP3 biology to accelerate neuroinflammation targeting in AD, and vice versa, exemplifies the added value that the cross-disease deposition framework provides over single-disease approaches. Anti-fibrotic therapies targeting TGF-β signaling may help limit end-organ remodeling across amyloidosis, glomerulonephritis, and atherosclerosis [207].

## Future directions and translational priorities

The cross-disease deposition framework developed in this review identifies several high-priority translational directions that could not have been recognized from any single-disease perspective. First, multi-omics integration, including genomics, transcriptomics, proteomics, lipidomics, and metabolomics, promises to map the molecular networks governing disease progression and to identify which individuals are most likely to transition from risk-factor exposure to overt deposition pathology [71, 208]. Single-cell and spatial transcriptomic approaches are already revealing disease-specific vulnerable cell states, including disease-associated microglia, lipid-droplet-accumulating microglia, and foam cell macrophages, that share transcriptional features and may respond to common intervention strategies [67, 109, 128]. Second, advanced imaging platforms that combine structural, metabolic, and molecular information, including PET-MRI approaches, will enable real-time longitudinal tracking of deposition kinetics and inflammatory activity in living patients [182–189]. These methods will be particularly valuable for defining when vascular and amyloid pathology first begin to converge and for identifying the most informative therapeutic windows. Third, systems biology network approaches that map shared nodes between atherosclerosis, AD, and related diseases, including NLRP3, APOE, TREM2, NF-κB, ABCA1, and lysosomal clearance pathways, may identify polypharmacological intervention points with cross-disease benefit. Rather than targeting each deposition disease in isolation, such approaches may reveal conserved regulatory bottlenecks whose modulation could favorably influence multiple disorders at once. Fourth, the emerging role of the gut microbiome in modulating systemic and neuroinflammation, lipid metabolism, and proteostasis represents an under-explored axis in deposition disease biology. Finally, therapeutic repurposing, applying agents developed for one deposition disease to mechanistically related conditions, represents a high-efficiency translational strategy: whether statins for neurovascular protection in AD, TTR stabilizers for cardiac ATTR amyloidosis, or NLRP3 inhibitors for combined cardiovascular and neuroprotection [101, 102, 134, 161, 175, 176]. Large-scale genomic studies will continue to identify novel targets applicable across the deposition disease spectrum [208].

## Conclusions

Pathological deposition of lipids, proteins, crystals, immune complexes, or metabolic substrates is a central, unifying mechanism across atherosclerosis, Alzheimer’s disease, systemic amyloidoses, immune complex–mediated nephropathies, crystal arthropathies, and lysosomal storage disorders. This review has argued that these conditions are mechanistically unified by shared failures of homeostasis: imbalance between production and clearance, structural transitions favoring aggregation or crystallization, microenvironmental extracellular matrix and ionic factors that nucleate and stabilize deposits, and chronic sterile inflammatory responses driven by NLRP3 inflammasome activation.

The added value of the cross-disease perspective developed here is threefold. First, it reveals molecular bridges that are often obscured by organ-based classifications: apoA-I dysfunction simultaneously mediates atherosclerosis through impaired reverse cholesterol transport and systemic amyloidosis through fibril formation; lipid-droplet-accumulating microglia in the aging AD brain parallel foam cell macrophages in atherosclerotic plaques; and NLRP3 inflammasome activation serves as a shared inflammatory effector linking cholesterol crystals, Aβ oligomers, urate crystals, and lysosomal damage across seemingly disparate diseases. Second, this framework identifies shared therapeutic targets, including proteostasis networks, phagocytic clearance mechanisms, the NLRP3 inflammasome, extracellular matrix interactions, and apolipoprotein biology, that may be leveraged for broadly applicable therapies. Third, it suggests specific opportunities for cross-disease translation: enhancing efferocytosis in atherosclerosis may inform strategies to restore microglial phagocytosis in AD; NLRP3 inhibitors validated in cardiovascular settings may be repurposed for neuroinflammation in AD; and apoA-I-based therapies may exert neuroprotective effects through Aβ solubilization.

Important knowledge gaps remain. These include the temporal and causal relationship between vascular and amyloid pathology in individual patients, the cell-type specificity of NLRP3 activation and its downstream effectors in each disease context, and the clinical translatability of preclinical phagocytosis-enhancing strategies. Addressing these gaps will require the integration of multi-omics, advanced imaging, artificial intelligence–assisted biomarker discovery, and systems biology approaches. Conceptualizing major cardiovascular, neurodegenerative, and systemic disorders as variations on a common deposition theme is not merely an intellectual exercise: it creates a framework for cross-disciplinary collaboration and therapeutic innovation with the potential to benefit patients across traditional disease boundaries.

## Data Availability

N/A.
